# Insufficient procedural anticoagulation during leadless pacing led to catheter-related thrombosis in a hemodialysis patient

**DOI:** 10.1186/s12872-021-02318-6

**Published:** 2021-10-18

**Authors:** Qiang Chen, Yong Jiang Ma, Chun Hong Zhang, Li Wei Zhang

**Affiliations:** Department of Cardiology, The Sixth Medical Center of Chinese PLA General Hospital, 6th, Fucheng Road, Haidian District, Beijing, 100853 People’s Republic of China

**Keywords:** Leadless pacemaker, Thrombosis, Anticoagulant, Hemodialysis, Case report

## Abstract

**Background:**

Leadless pacemaker was a promising innovation than traditional transvenous pacemaker, the procedural complications were prone to be bleeding-related. However, very few reports also concerned about the thrombus formation during the procedure.

**Case presentation:**

A hemodialysis patient with diabetic gangrene of right foot suffered from catheter-related thrombosis during leadless pacing, resulting in failure of recapture the pacemaker. A low activated clotting time (ACT) level of 104 s confirmed the insufficiency of anticoagulation. Finally, the whole delivery catheter had to be removed from the delivery sheath, another new pacemaker system was applied and successfully implanted after adjusting the ACT level to 248 s.

**Conclusion:**

Catheter-related thrombosis could be a large obstacle for leadless pacemaker implantation. In addition to routine anticoagulation, ACT monitoring might be necessary during the procedure.

**Supplementary Information:**

The online version contains supplementary material available at 10.1186/s12872-021-02318-6.

## Background

Leadless pacemaker was a promising innovation than traditional transvenous pacemaker with its miniaturized profile and superior safety. The procedural complications such as venous site hemorrhage, arteriovenous fistulas and pericardial effusion had been studied [1], which were prone to be bleeding-related. However, very few reports also concerned about the thrombus formation during the procedure. We presented a case for the first time that a hemodialysis patient with diabetic gangrene of right foot suffered from catheter-related thrombosis, resulted in difficulty of recapture and withdrawal the pacemaker. A low activated clotting time (ACT) level of 104 s confirmed the insufficiency of anticoagulation.

## Case presentation

**Fig. 1:**
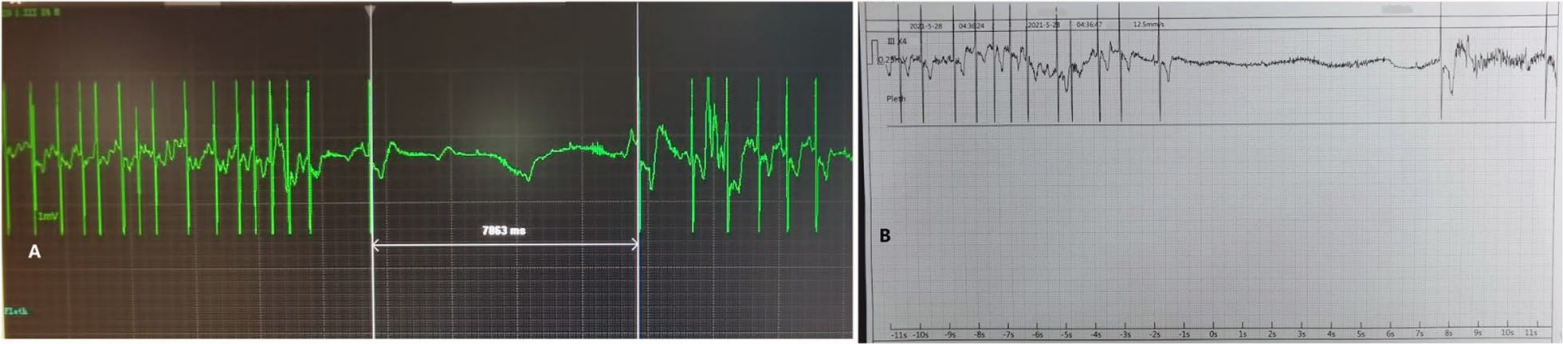
**a** ECG showed paroxysmal atrial fibrillation and sudden sinus arrest for up to 7.8 s. **b** Another ECG showed paroxysmal atrial fibrillation with long RR interval for up to 9 s

A 68-year-old man, who was 175 cm height and 75 kg weight with a body mass index (BMI) of 24.5, suffered from syncope for several times, the ECG showed paroxysmal atrial fibrillation with long RR intervals for up to 9.3 s (Fig. 1). The echocardiography showed mild-to-moderate tricuspid regurgitation, normal ventricular wall motion and normal left ventricular ejection fraction. The medical history included end-stage renal disease (ESRD) requiring hemodialysis for 6 years, diabetic gangrene of right foot for 5 months. Indication for pacemaker implantation was established according to the ECG results, in order to prevent transvenous pocket inflammation or systemic inflammation from the gangrenous foot, the Micra leadless pacemaker (Medtronic, Inc. Minneapolis, USA) was recommended and the informed consent was approved by the patient.

The right femoral vein was successfully punctured and inserted with a 6 French sheath, and a bolus of 3000 IU unfractionated heparin (UFH) was intravenous administrated at the beginning of the procedure. The access site was gradually dilated by 8- and 12-French dilators, and a 23-French delivery sheath was introduced into the inferior vena cava, then the pacemaker delivery catheter was connected to the sheath. All delivery devices were flushed and connected with heparinized saline properly. The Micra pacemaker was advanced and conducted into the mid-septum of right ventricle and the fluoroscopic location was acceptable. However, electrical measurement showed a low sensing amplitude of only 2.8 mV, suggesting that the pacemaker needed to be repositioned again.

**Fig. 2:**
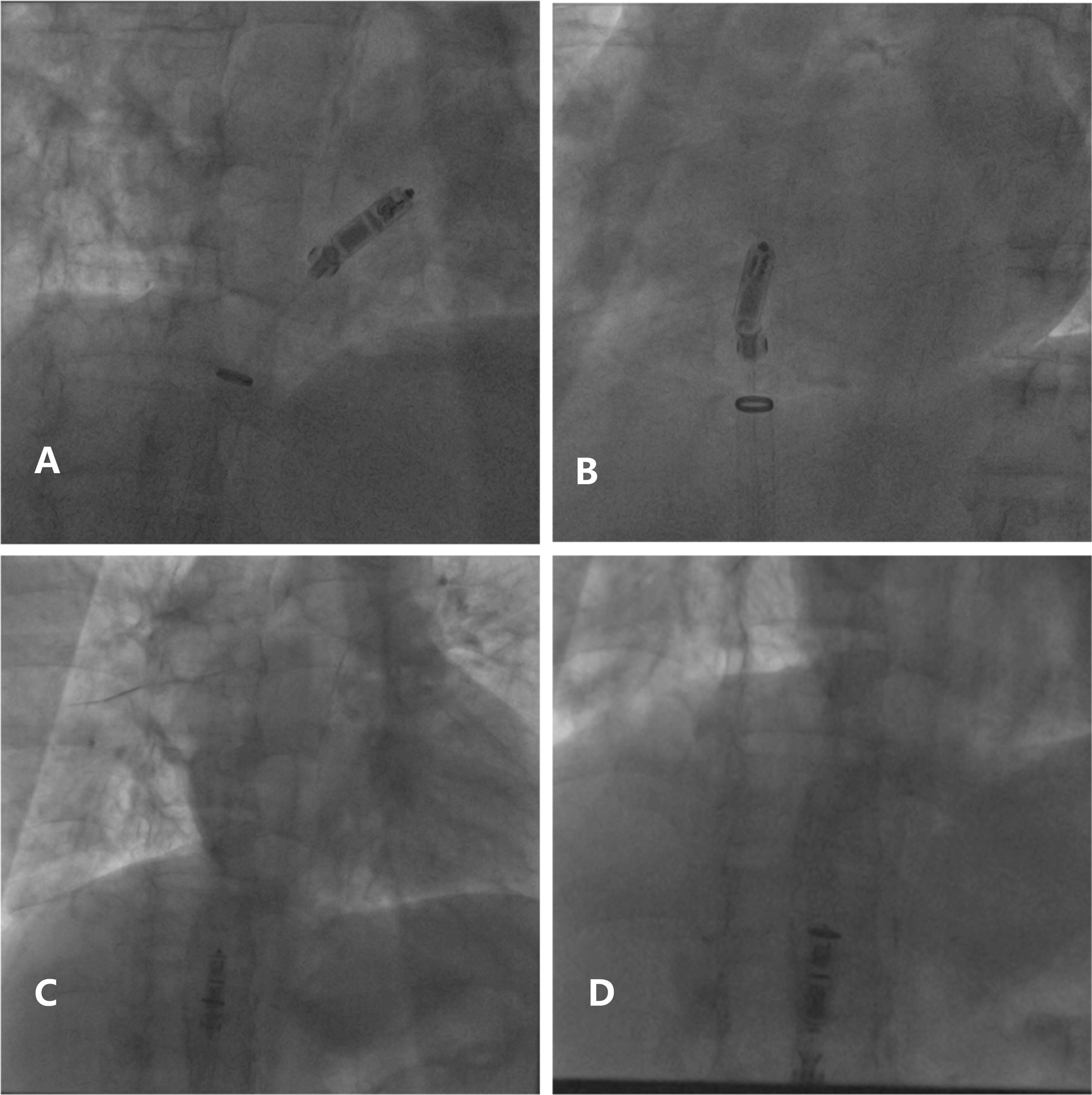
**a** Fluoroscopic RAO angle showed the pacemaker was coaxial with the delivery cup but could not be pulled back. **b** Fluoroscopic LAO angle showed the recapture of pacemaker failed again. **c** The whole delivery catheter was withdrawing back into the delivery sheath. **d** The pacemaker was recaptured with great resistance

For recapture, the tether connected to the handle was pulled with constant tension and the pacemaker was closed to the recapture cone, different fluoroscopic angles were viewed to confirm the devices were coaxial. Since then the delivery cup could not be advanced due to great resistance, the pacemaker was not able to be withdrew into the catheter (Fig. 2, Additional file 1). Finally, the whole delivery catheter had to be removed from the delivery sheath (Fig. 2, Additional file 2).

**Fig. 3:**
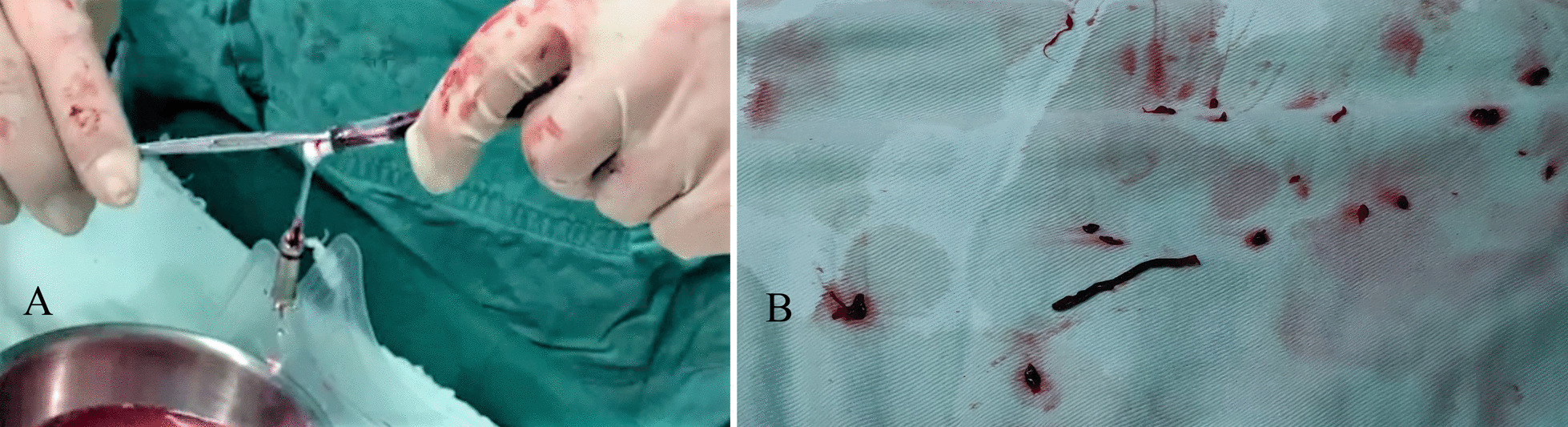
**a** The operator tried to clean the thrombi within the delivery cup. **b** Thrombi removed from the delivery catheter

**Fig. 4:**
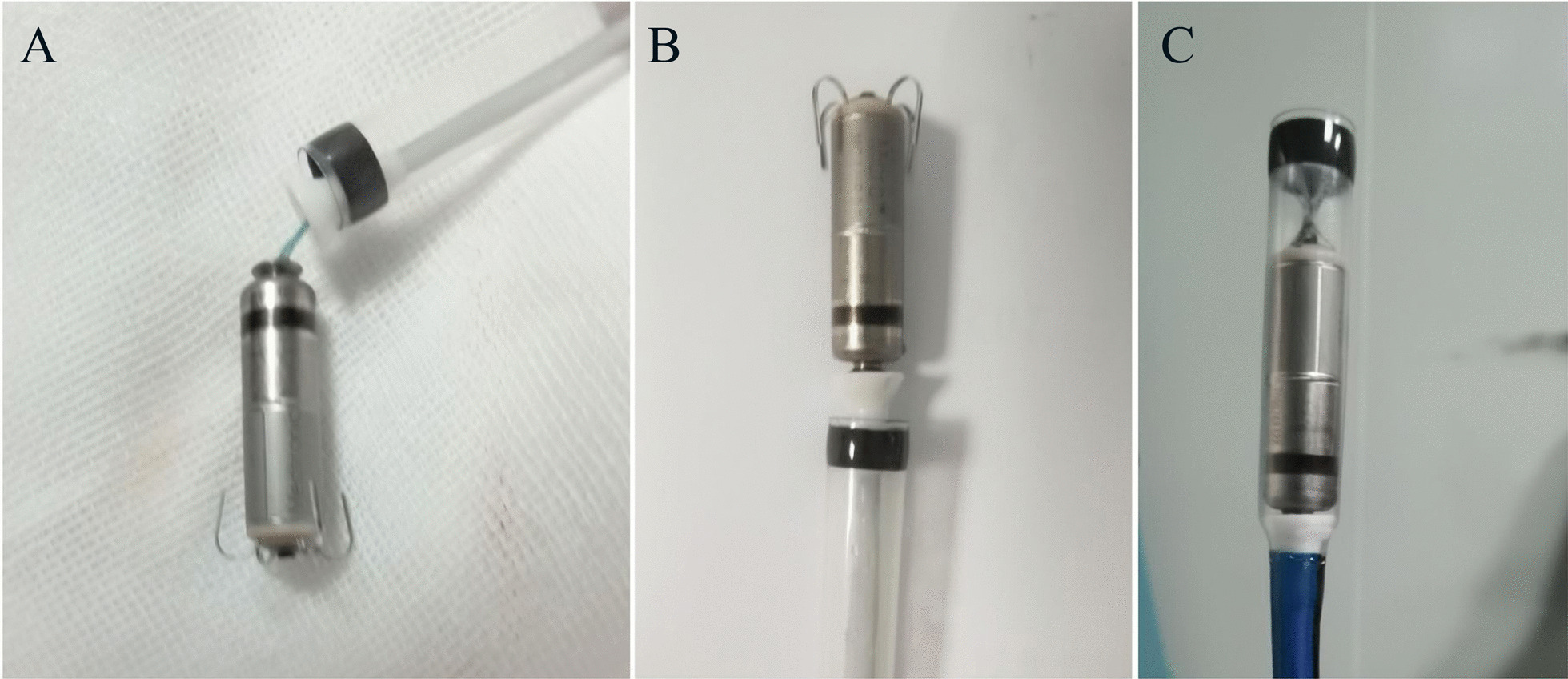
**a, b** After being removed all the thrombi the structure of the delivery system remained integrity. **c** The pacemaker was recaptured without difficulty

Luckily no valvular or myocardial tissues were found at the tip of the catheter, instead the lumen was fully filled with heavy thrombi (Fig. 3). An instant ACT level of 104 s revealed that procedural anticoagulant therapy was insufficient, thus, additional 4000 IU (a total amount of 7000 IU) UFH was administrated immediately and the ACT level turned to 248 s 10 min later. After being removed all the thrombi the structure and mobility of the delivery catheter recovered (Fig. 4), which in turn proved that the failure of recapture was caused by the thrombi within the lumen. For preventing thromboembolism, a new pacemaker system was applied and successfully implanted. The final electrical measurement was satisfied: pacing threshold was 0.5 V at 0.24 ms, sensing amplitude was 7.9 mV, and pacing impedance was 560 Ω. The final ACT level was 204 s.

## Discussion

An increasing number of patients will no doubt benefit from the new pacing technology. The procedure seems to be easier and safer than traditional pacing with over 95% successful rate. The key point of the implantation is to settle the pacemaker with suitable electrical parameters, which sometimes requires retrieving and redeploying the device. In most of the time the retrieval is without difficulty unless the nearby myocardial tissues are hooked by the pacemaker. In this case, we proved that thrombosis within the catheter could also hinder the movement of delivery cup or somehow affect the coaxality and led to the failure of recapture.

According to the guideline on percutaneous coronary intervention UFH is the preferred anticoagulant [2], the recommended dose is 70–100 U/kg intravenous and need not to be adjusted in CKD patients. But it is not clear whether routine anticoagulation is mandatory during leadless pacemaker implantation. Thrombosis s very rare [3] as compared with the bleeding complications, some experienced centers recommended a dose of 3000–5000 IU UFH intravenous without monitoring the ACT [4] and continuously flushing the delivery devices with heparinized saline. But in hemodialysis patients the data was lacking.

In our case the patient was at both high thromboembolic and bleeding risks with 4 points of CHA2DS2VASc score and 3 points of HASBLED score, which made the strategy for anticoagulation challenging. Considering the hemodialysis background UFH seemed to be the optimal anticoagulant for the patient. But apparently 3000 IU UFH was insufficient according to the first ACT level, additional 4000 IU UFH turned the level into 248s which almost reached the ideal ACT level during coronary intervention (between 250s and 300s) and successfully prevented another thrombosis complication. This could be an important finding: in complex situations, ACT levels should be measured in order to evaluate the anticoagulant effects in time. But the proper ACT ranges for leadless pacemaker implantation were still unknown and needed to be discussed further.

The safety during periprocedural period and the 1-year follow-up was promising for hemodialysis patient [5]. The patient also recovered well and was discharged 3 days later. Post-procedural echocardiography showed no evidence of pericardial effusion.

In conclusion, Catheter-related thrombosis could be a large obstacle for leadless pacemaker implantation. In addition to routine anticoagulation, ACT monitoring might be necessary during the procedure, and the appropriate ACT levels deserve further study.

## Supplementary Information

**Additional file 1**. Failure of withdrawing the pacemaker into the catheter, the delivery cup could not be advanced due to great resistance.

**Additional file 2**. The whole delivery catheter was removed from the delivery sheath.

## Data Availability

All data generated or analyzed during this study are included in this published article [and its Additional files 1, 2].

## Abbreviations

ACT: Activated clotting time
BMI: Body mass index
ESRD: End-stage renal disease
UFH: Unfractionated heparin
